# Supplementary figures and images for: SARS-CoV-2 Omicron subvariant genomic variation associations with immune evasion in Northern California: A retrospective cohort study
Source: PLoS One. 2025 Feb 24;20(2):e0319218. doi: 10.1371/journal.pone.0319218 (PMC11849856; doi:10.1371/journal.pone.0319218)

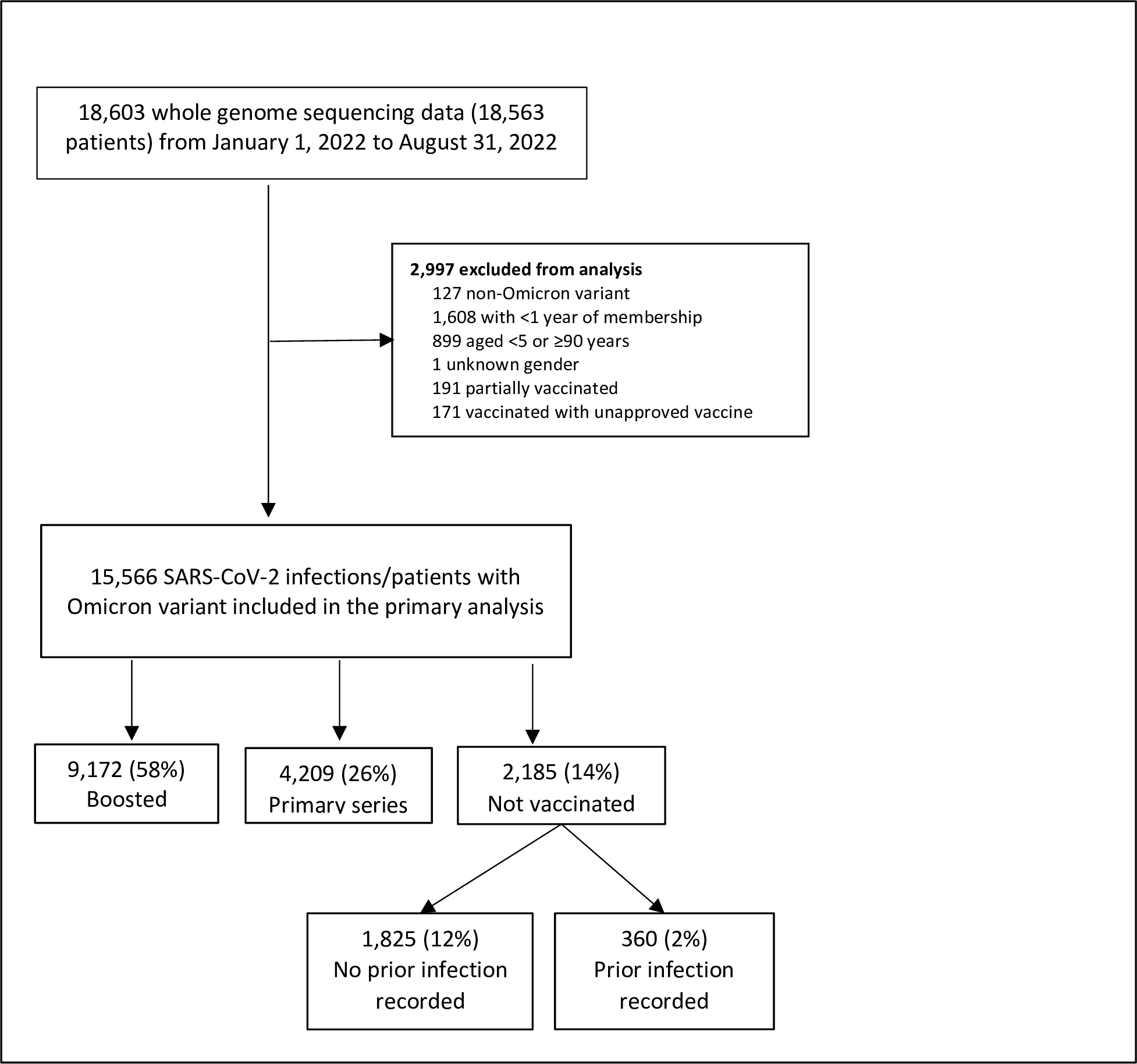

Supplement: S1 Fig — Cohort eligibility flowchart with prior recorded infection and vaccination status. (TIF) [file pone.0319218.s001.tif]

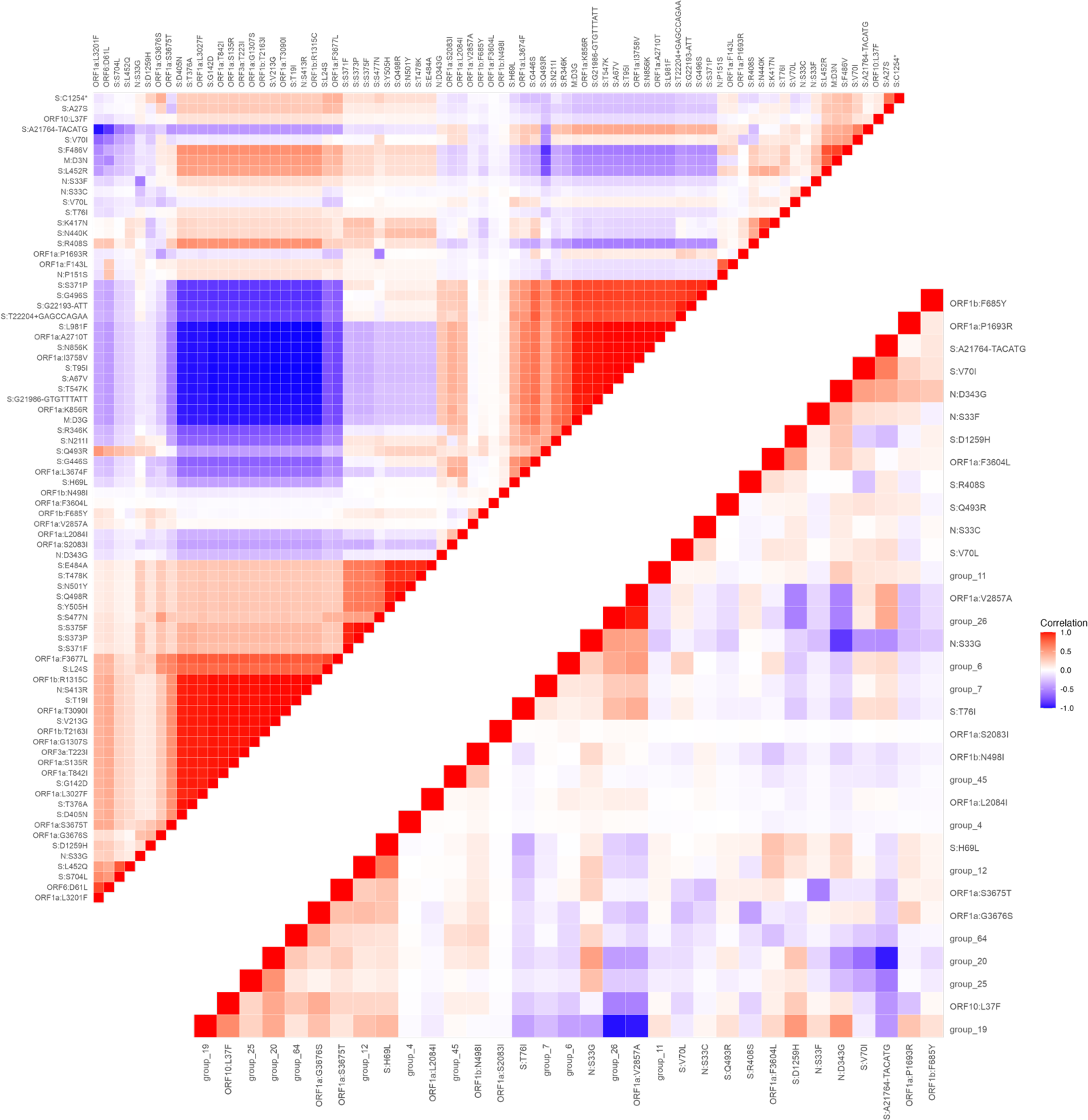

Supplement: S2 Fig — Pearson correlations of SNP presence in specimens, before and after clustering. (TIF) [file pone.0319218.s002.tif]

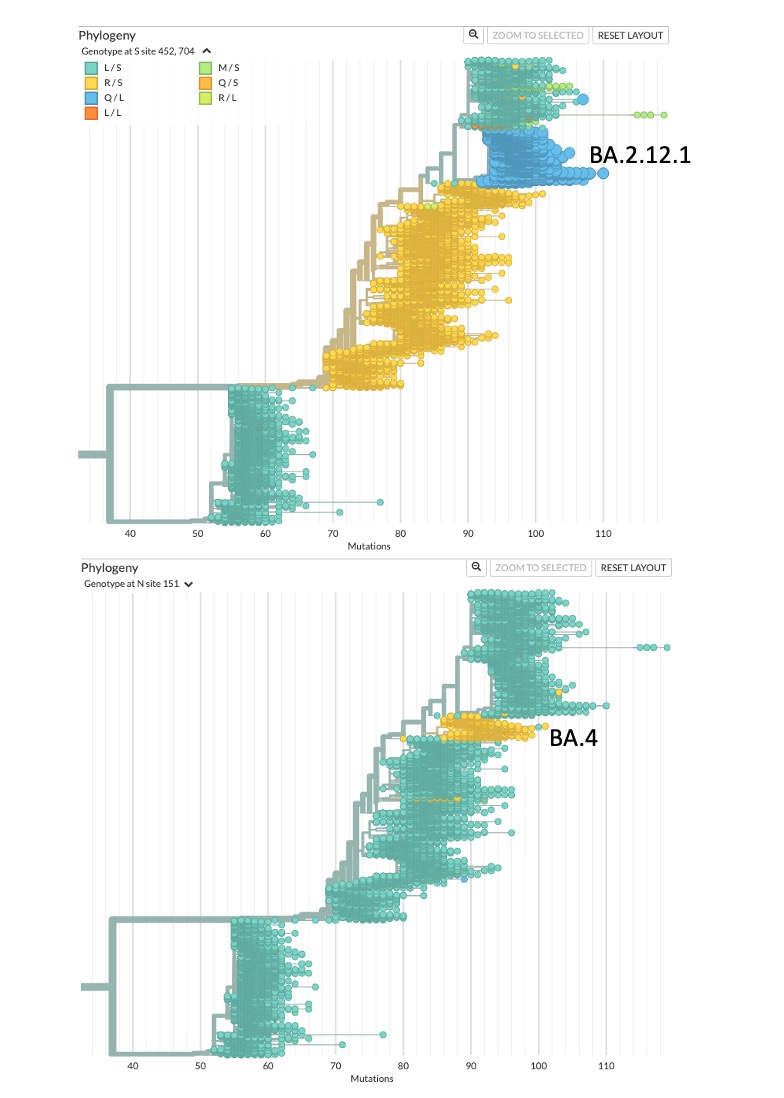

Supplement: S3 Fig — Phylogenies of 15,045 Omicron genomes colored by group. Mutations in group 52 (top) represent a clade defined by the BA.2.12.1 subvariant (in blue). Mutations in group 4 (bottom) fall into a subclade of BA.4 (in yellow). (TIF) [file pone.0319218.s003.tif]

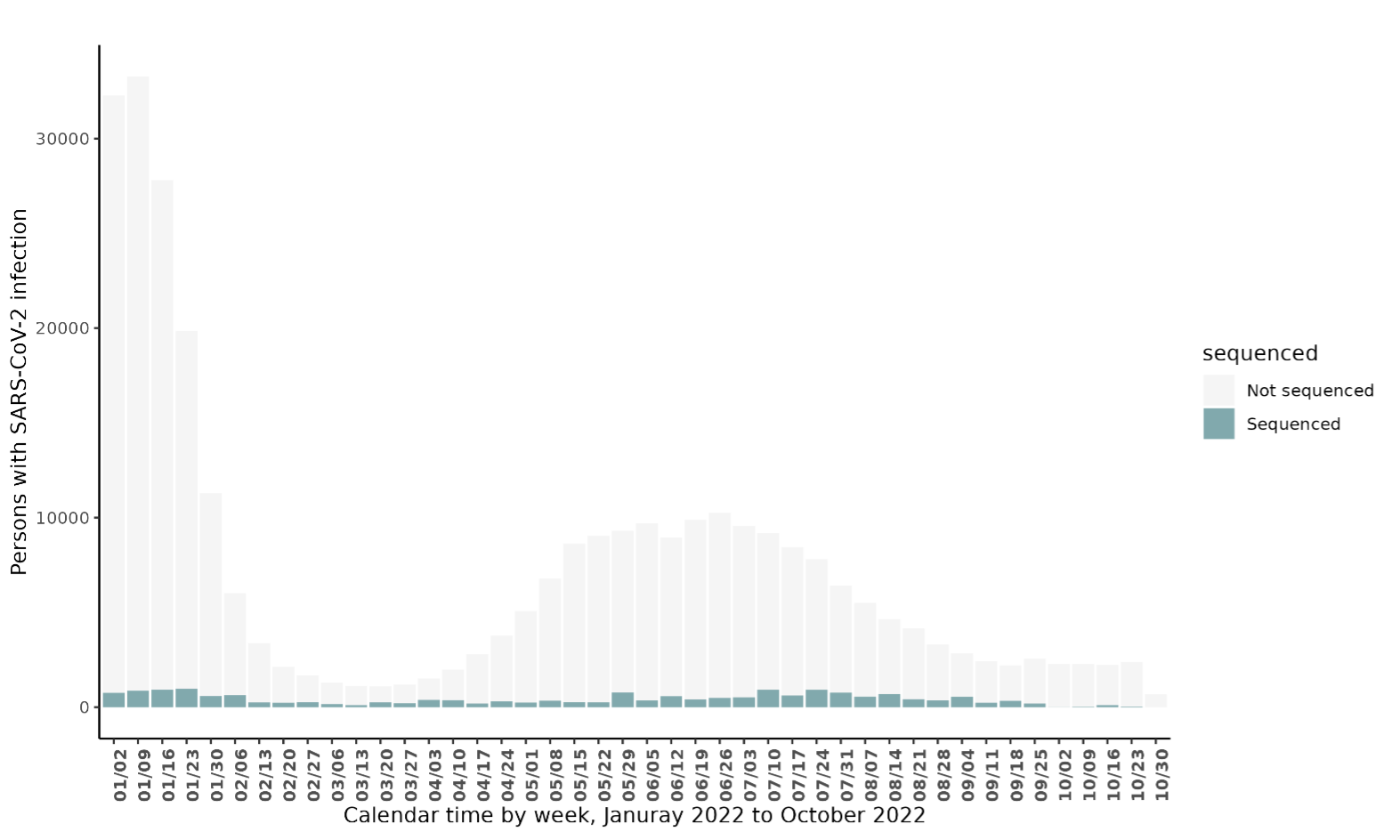

Supplement: S4 Fig — Persons with recorded SARS-CoV-2 infection at KPNC by week from January 1, 2022 to October 31, 2022. (TIF) [file pone.0319218.s004.tif]

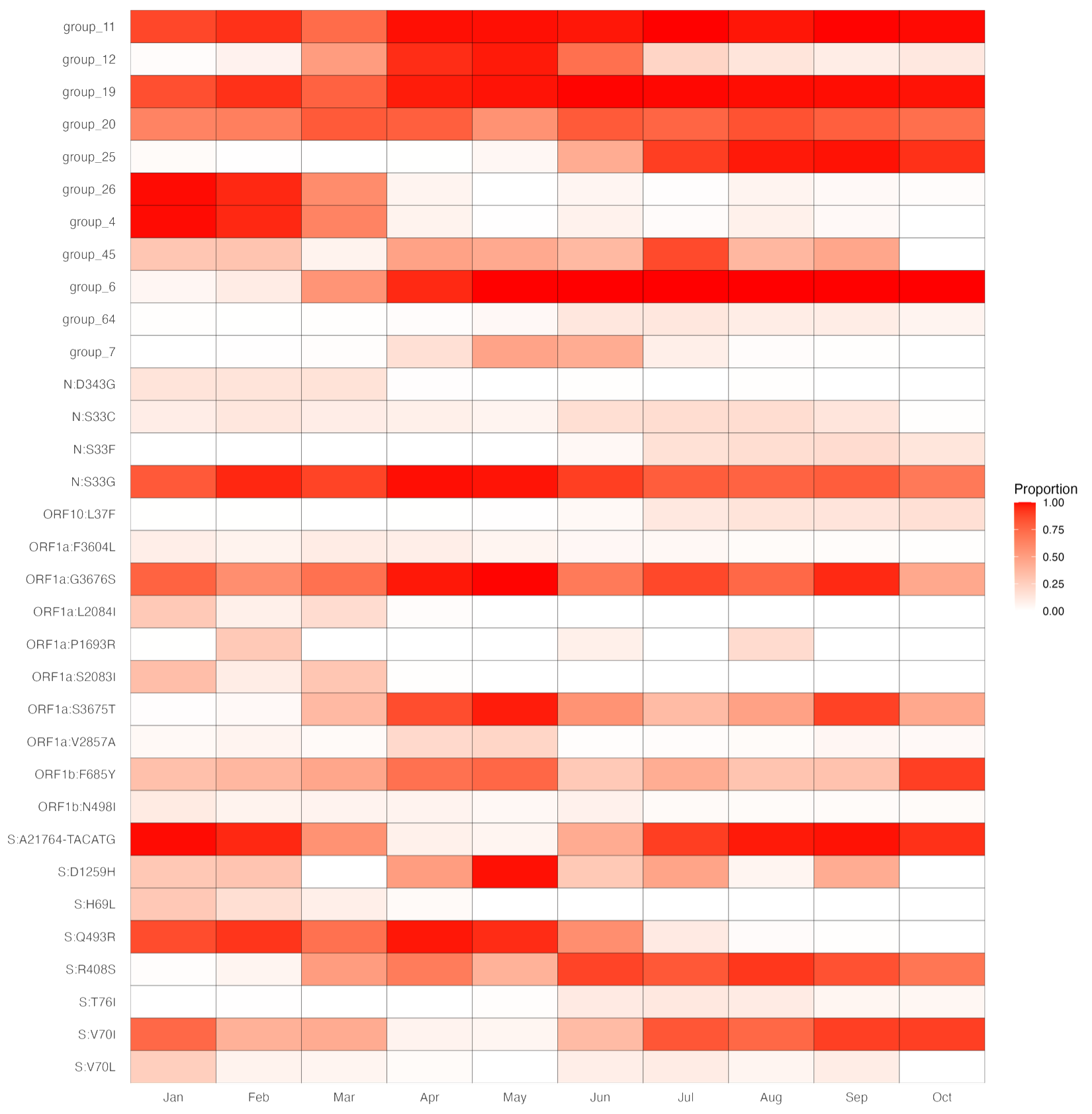

Supplement: S5 Fig — Proportion of specimens with a given SNP or group, by month, January–October 2022. (TIF) [file pone.0319218.s005.tif]

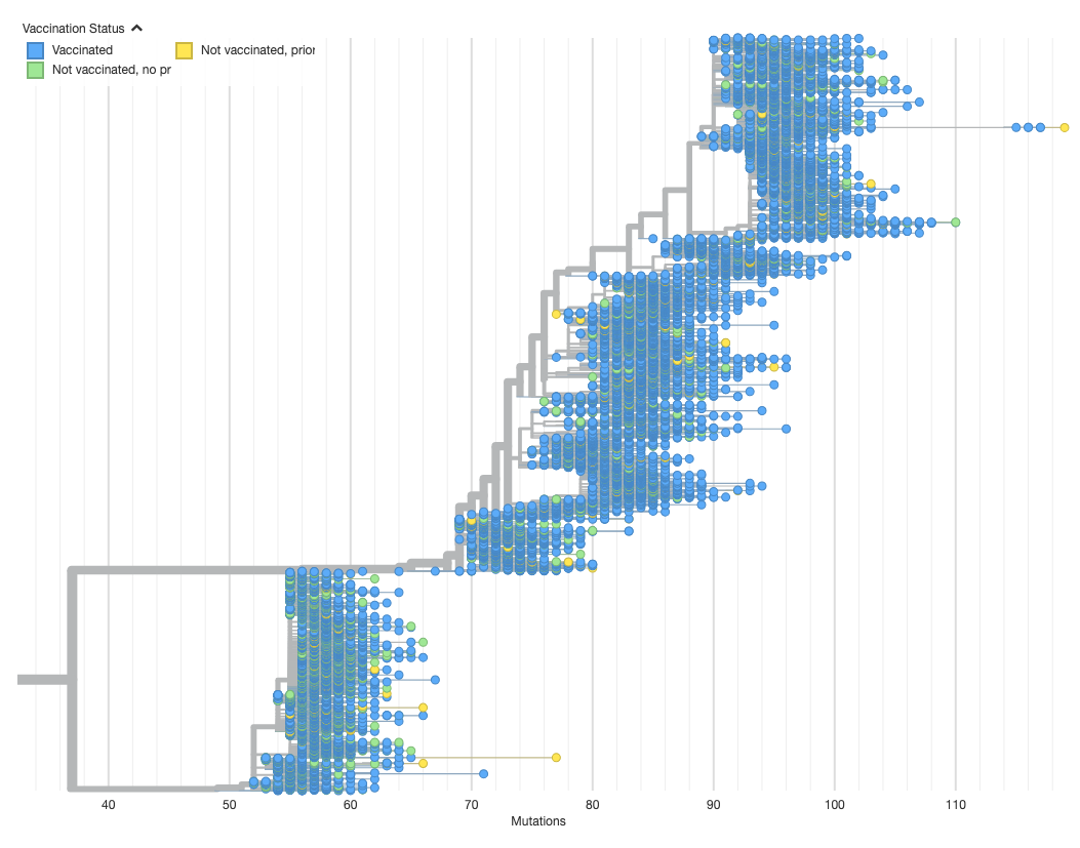

Supplement: S6 Fig — Phylogeny of 15,045 Omicron genomes sampled between 1/1/2022 and 9/25/22. Branch lengths represent divergence from Wuhan reference genome and nodes are colored by vaccination status. Vaccinated category includes primary series and primary plus booster. (TIF) [file pone.0319218.s006.tif]

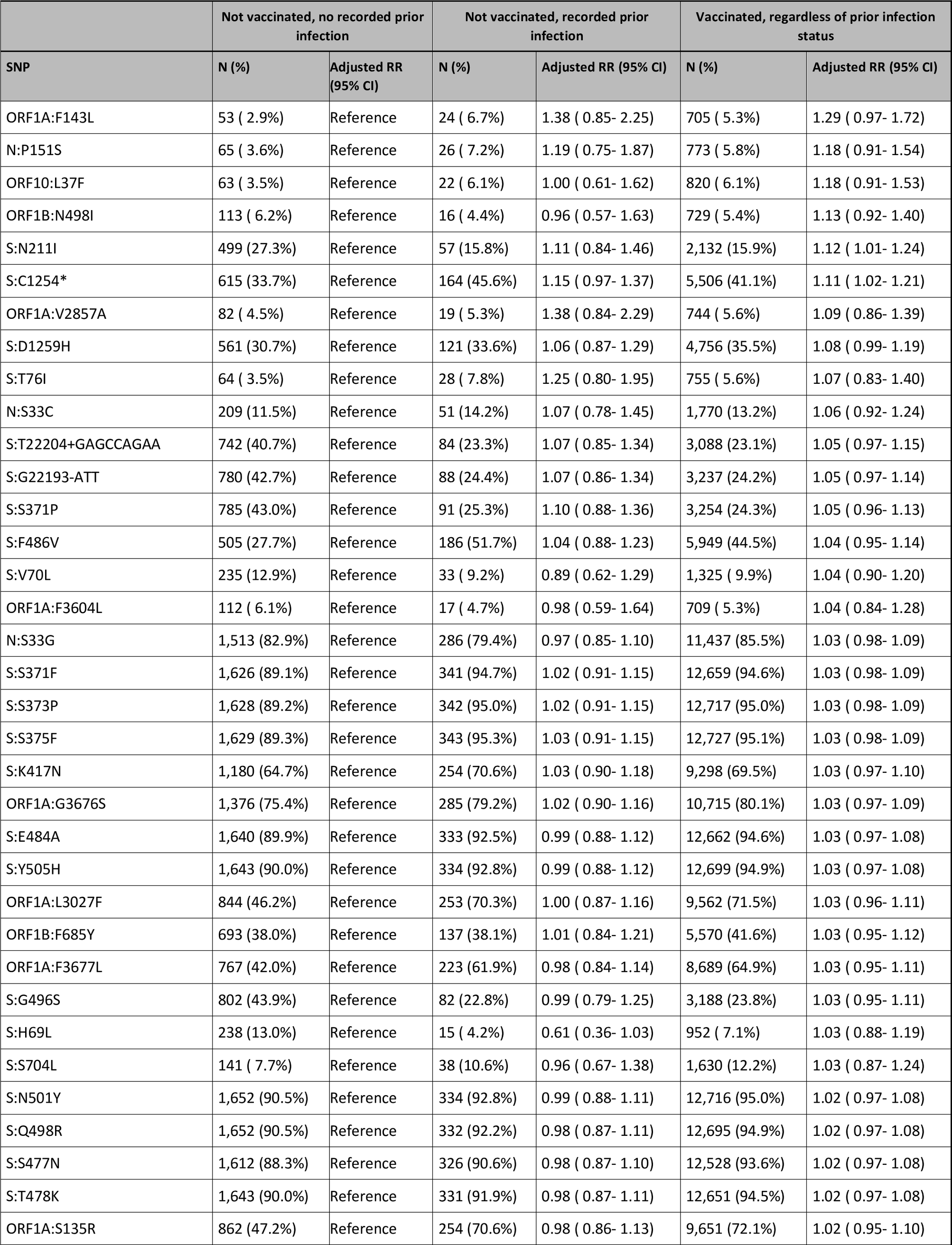

Supplement: S1 Table — Association between SNP presence and vaccine status (pooling over complete and boosted) among persons included in this analysis with SARS-CoV-2 infection with Omicron variant in Northern California, January 1, 2022 - October 31, 2022 (N = 15,566). (TIF) [file pone.0319218.s007.tif]

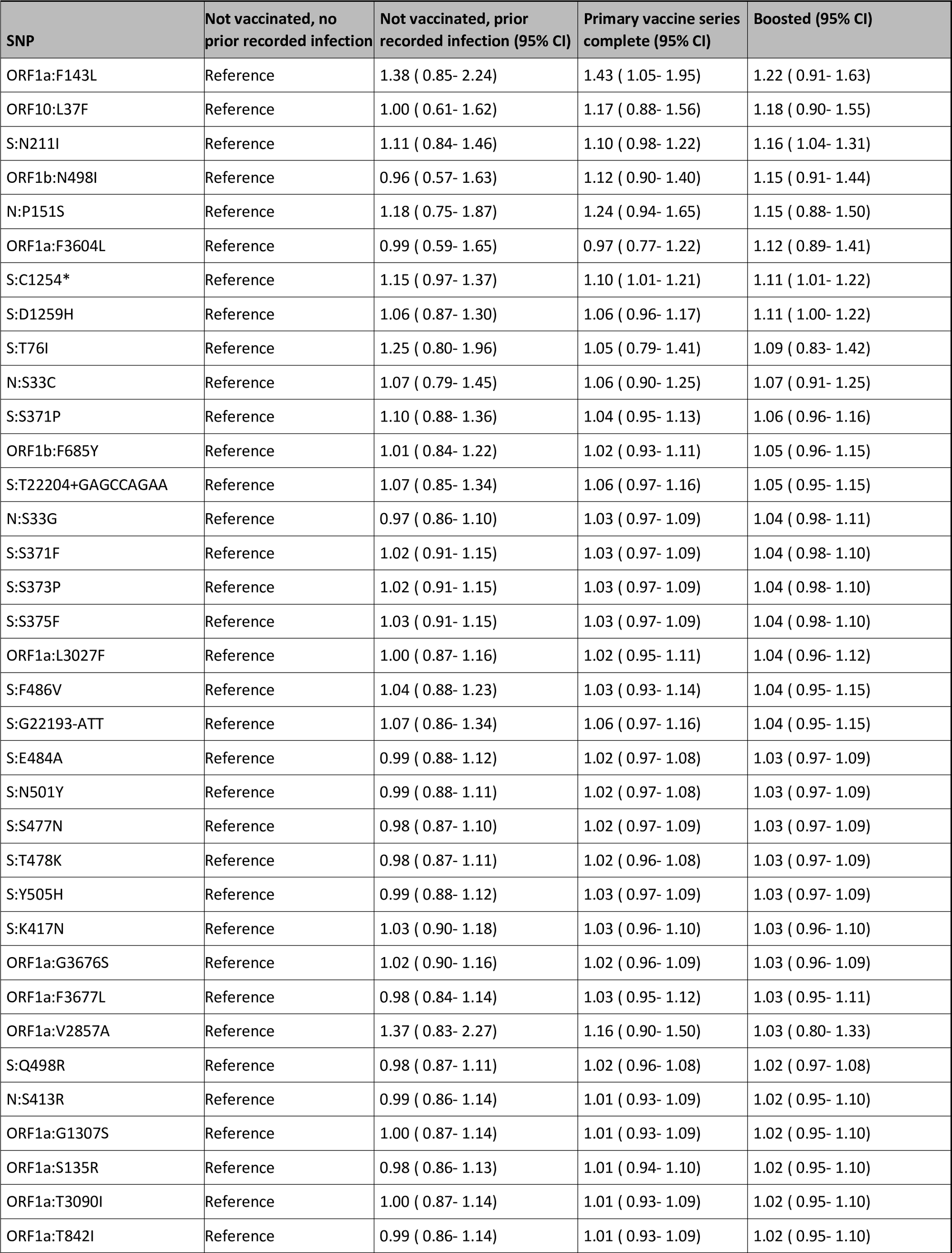

Supplement: S2 Table — Association between SNP presence and vaccine status (separating out complete and boosted) among persons included in this analysis with SARS-CoV-2 infection with Omicron variant in Northern California, January 1, 2022 – October 31, 2022 (N = 15,566). (TIF) [file pone.0319218.s008.tif]

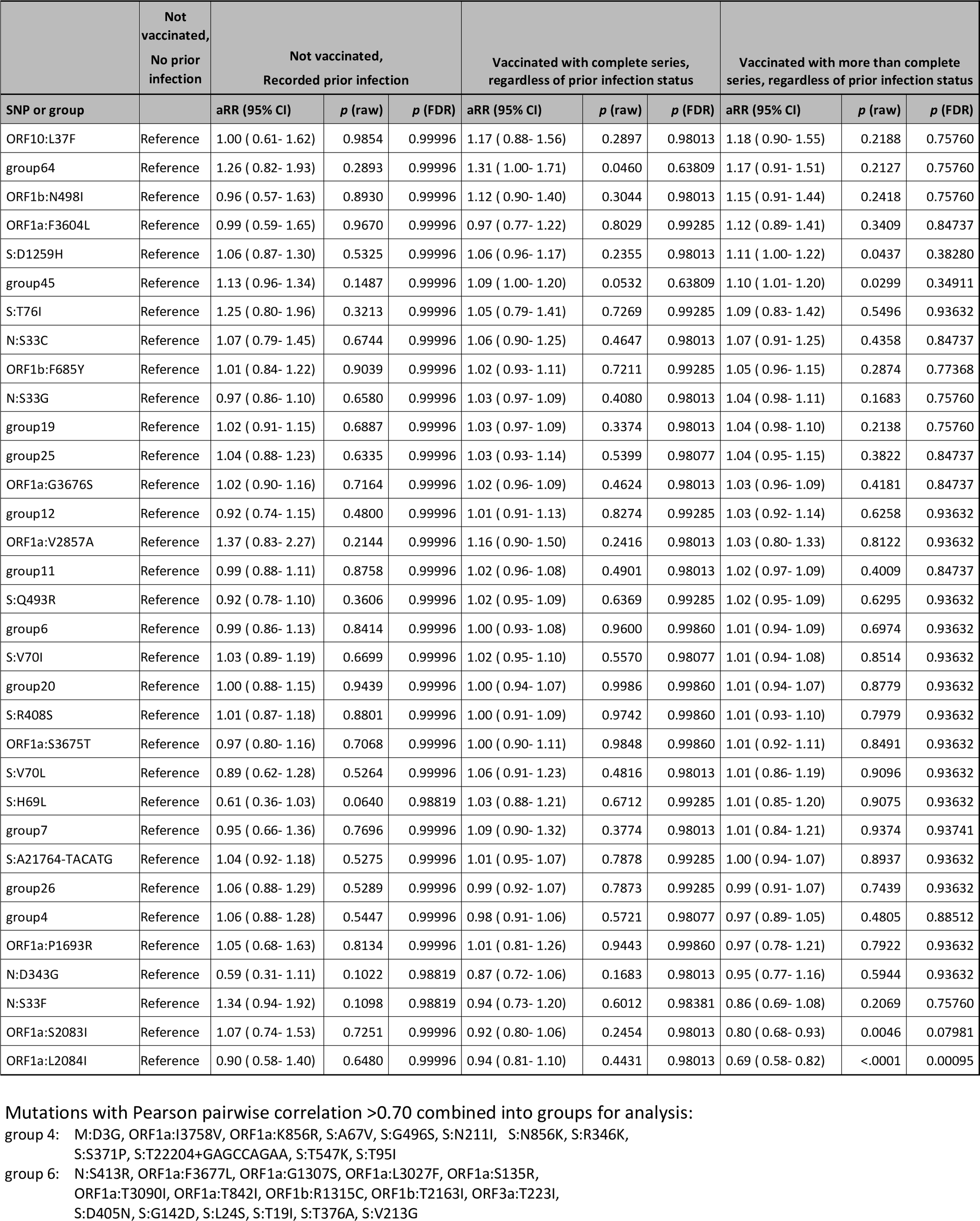

Supplement: S3 Table — Association between mutation groups and vaccine status (separating complete and boosted) among persons included in this analysis with SARS-CoV-2 infection with Omicron variant in Northern California, January 2022–October 2022 (N = 15,566). Adjusted risk ratios derived from Poisson regression models to assess the association of immunity status with each mutation group adjusting for age, sex, race/ethnicity, Charlson comorbidity index score, and month of SARS-CoV-2 infection. (TIF) [file pone.0319218.s009.tif]
