# Supplementary material for: SARS-CoV-2 Omicron subvariant genomic variation associations with immune evasion in Northern California: A retrospective cohort study
Source: PLoS One. 2025 Feb 24;20(2):e0319218. doi: 10.1371/journal.pone.0319218 (PMC11849856; doi:10.1371/journal.pone.0319218)
Supplement: Attachment — Submitted filename: Nugent_Omicron_Response_To_Reviewers.docx [file pone.0319218.s010.docx]

1. Please note that funding information should not appear in any section or other areas of your manuscript. We will only publish funding information present in the Funding Statement section of the online submission form. Please remove any funding-related text from the manuscript.

**Response: We have removed all references to funding from the manuscript.**

2. In this instance it seems there may be acceptable restrictions in place that prevent the public sharing of your minimal data. PLOS requires that the minimal data for your study be made available upon request in this case. However, in line with our goal of ensuring long-term data availability to all interested researchers, PLOS’ Data Policy states that authors cannot be the sole named individuals responsible for ensuring data access. Data requests to a non-author institutional point of contact, such as a data access or ethics committee, helps guarantee long term stability and availability of data. Providing interested researchers with a durable point of contact ensures data will be accessible even if an author changes email addresses, institutions, or becomes unavailable to answer requests.

1) If there are ethical or legal restrictions on sharing a de-identified data set, please explain the applicable restrictions in detail (e.g., data contain potentially identifying or sensitive patient information) and who has imposed them (e.g., a Research Ethics Committee or Institutional Review Board, etc.).

**Response: The data are both potentially identifying and contain sensitive health information. Our IRB has not allowed us to share the data outside of our institution unless the following criteria/procedures have been met: *Anonymized data that support the findings of this study may be made available from the investigative team in the following conditions: (1) agreement to collaborate with the study team on all publications, (2) provision of external funding for administrative and investigator time necessary for this collaboration, (3) demonstration that the external investigative team is qualified and has documented evidence of training for human subjects protections, and (4) agreement to abide by the terms outlined in data use agreements between institutions.***

2) Please also provide non-author contact information (phone/email/hyperlink) for a data access committee, ethics committee, or other institutional body to which data requests may be sent.

**Response: Requests may be sent to the KPNC IRB:** [**https://wiki.kp.org/display/irb/**](https://wiki.kp.org/display/irb/)**, 866-241-0690, kpnc.irb@kp.org**

3) If applicable, please also provide any necessary information which interested researchers would need when requesting access to data in order to obtain the minimal data set for your study.

**Response: See response to item #1, which discusses the criteria we would require for interested researchers.**
